# Risk of radiation-induced second malignant neoplasms from photon and proton radiotherapy in paediatric abdominal neuroblastoma

**DOI:** 10.1016/j.phro.2021.06.003

**Published:** 2021-07-09

**Authors:** Sophie Taylor, Pei Lim, Reem Ahmad, Ammar Alhadi, William Harris, Vasilis Rompokos, Derek D'Souza, Mark Gaze, Jennifer Gains, Catarina Veiga

**Affiliations:** a Centre for Medical Image Computing, Department of Medical Physics and Biomedical Engineering, University College London, London, UK; b Department of Oncology, University College London Hospitals NHS Foundation Trust, London, UK; c Radiotherapy Physics Services, University College London Hospitals NHS Foundation Trust, London, UK

**Keywords:** Childhood cancer, Paediatric, Abdominal neuroblastoma, Second malignant neoplasm, Proton therapy, Pencil beam scanning, Intensity modulated arc therapy (IMAT)

## Abstract

**Background and Purpose:**

State-of-the-art radiotherapy modalities have the potential of reducing late effects of treatment in childhood cancer survivors. Our aim was to investigate the carcinogenic risk associated with 3D conformal (photon) radiation (3D-CRT), intensity modulated arc therapy (IMAT) and pencil beam scanning proton therapy (PBS-PT) in the treatment of paediatric abdominal neuroblastoma.

**Materials and Methods:**

The risk of radiation-induced second malignant neoplasm (SMN) was estimated using the concept of organ equivalent dose (OED) for eleven organs (lungs, rectum, colon, stomach, small intestine, liver, bladder, skin, central nervous system (CNS), bone, and soft tissues). The risk ratio (RR) between radiotherapy modalities and lifetime absolute risks (LAR) were reported for twenty abdominal neuroblastoma patients (median, 4y; range, 1-9y) historically treated with 3D-CRT that were also retrospectively replanned for IMAT and PBS-PT.

**Results:**

The risk of SMN due to primary radiation was reduced in PBS-PT against 3D-CRT and IMAT for most patients and organs. The RR across all organs ranged from 0.38 ± 0.22 (bladder) to 0.98 ± 0.04 (CNS) between PBS-PT and IMAT, and 0.12 ± 0.06 (rectum and bladder) to 1.06 ± 0.43 (bone) between PBS-PT and 3D-CRT. The LAR for most organs was within 0.01–1% (except the colon) with a cumulative risk of 21 ± 13%, 35 ± 14% and 35 ± 16% for PBS-PT, IMAT and 3D-CRT, respectively.

**Conclusions:**

PBS-PT was associated with the lowest risk of radiation-induced SMN compared to IMAT and 3D-CRT in abdominal neuroblastoma treatment. Other clinical endpoints and plan robustness should also be considered for optimal plan selection.

## 1 Introduction

Neuroblastomas are the most common extracranial solid tumour in children [1], [2]. In 80% of cases, malignancy arises from cells of the adrenal medulla and paravertebral ganglia of the abdomen [3]. Most patients present with disseminated disease at diagnosis; in this high-risk group, radiotherapy to the primary tumour bed plays a key role in disease management alongside chemotherapy, surgery and immunotherapy [4], [5], [6], [7], [8], [9]. Three-dimensional Conformal (photon) Radiation Therapy (3D-CRT) with opposing anterior and posterior parallel (AP/PA) fields is still the standard radiotherapy modality choice in many centres, despite full prescription delivery being frequently hindered by the low tolerance dose of the neighbouring liver and kidneys [10], [11]. Advanced state-of-the-art modalities such as Intensity Modulated Arc Therapy (IMAT) and proton therapy have great potential to address challenges in neuroblastoma treatment [12], [13] due to their increased conformity and reduced non-target high-dose regions [11], [14], [15], [16], [17], [18]. However, adopting new radiotherapy modalities in paediatric cancer management has historically been approached with caution due to the potential impact of long-term sequelae [19]. Widespread adoption of IMAT in place of 3D-CRT was slow due to concerns that the associated low-dose radiation “bath” may increase patient’s carcinogenic risk, despite the more conformal high-dose volume. The risk of radiation-induced second malignant neoplasms (SMNs) poses a real threat to childhood cancer survivors and radiotherapy is a recognised risk-factor of carcinogenesis [20], [21]. Risks associated with newer modalities must be confirmed on long-term patient follow-up data. This is challenging since SMNs after radiotherapy have a latency period of 10 years or more after exposure, and hence several decades of comprehensive data collection are required [22], [23], [24].

Investigating the relative safety of newer radiotherapies in terms of carcinogenic risk is of utmost importance in childhood cancer management. Due to challenges associated with epidemiological studies, in silico treatment planning evaluations of radiation-induced SMN risk are an attractive alternative for estimating the long-term risks of newer treatment approaches [16], [25], [26]. While SMN risk evaluations have been previously conducted in the abdominal neuroblastoma cohort, existing studies are not comprehensive [16], [17], [26]. The risks were usually estimated for a small number of patients using variable methodologies [27], [28] and previous studies also did not focus on increasingly popular modalities such as IMAT and Pencil Beam Scanning Proton Therapy (PBS-PT).

Therefore, this study aimed to perform a comprehensive evaluation of the theoretical benefits of PBS-PT versus IMAT and 3D-CRT with respect to carcinogenic risk in eleven organs for paediatric abdominal neuroblastoma.

## 2 Materials and methods

### 2.1 Patient selection

Twenty high-risk patients with abdominal primaries who received 3D-CRT historically at University College London Hospital were selected for this study as described in our previous publications [11], [15]. Consent for data usage for research purposes was available for all cases. The datasets were provided fully anonymised. The male to female ratio of this cohort was 11:9 with a median age of 4 years (range; 1–9 years). Nine patients displayed midline tumours where the remaining presented with well-lateralised tumours (five right-sided and six left-sided) [15].

### 2.2 SMN-relevant organs

The SMN risk associated with each radiotherapy modality was assessed for eleven organs: the lungs, rectum, colon, stomach, small intestine, liver, bladder, skin, central nervous system (CNS), bone, and soft tissues. All organs-at-risk were manually delineated on planning CT images and reviewed by a clinical expert. The skin was defined as a 2 mm shell of the outer body contour. The bones were extracted by applying a threshold (>150HU) within the body while excluding high-intensity artefacts. The remaining regions were combined into the soft tissues.

The CT images covered the abdomen and thorax only. Volumes outside this field-of-view were estimated by rigidly aligning each CT with an age- and sex-specific XCAT phantom [29], [30]. A total of 18 XCAT models aged 1–9 years for each sex, generated with default settings, were available. Each patient was matched to its most similar phantom automatically, considering age and body contour similarity. Comparable segmentations were generated on all phantoms and used to estimate the missing anatomy (head and limbs). The radiotherapy dose was assumed to be zero outside the imaging field-of-view.

### 2.3 Overview of treatment planning techniques

**Fig. 1:**
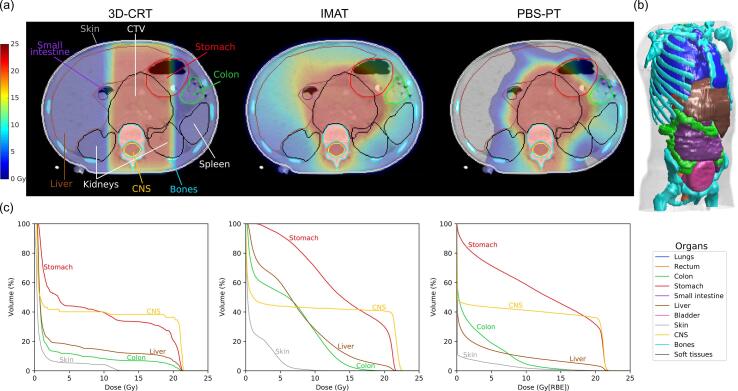
Example of (a) CT and overlaid anatomy and doses distributions for 3D-CRT, IMAT and PBS-PT plans, (b) volume rendering of the eleven organs included in the risk assessment analysis (lungs, rectum, colon, stomach, small intestine, liver, bladder, skin, central nervous system (CNS), bone, and soft tissues), and (c) dose-volume histograms (DVHs) for selected organs. Note that the vertebra is homogeneously irradiated to reduce growth asymmetry. Colour version online.

Dose distributions from clinically acceptable 3D-CRT, IMAT and PBS-PT plans, described in previous studies were analysed [11], [15]. The prescribed dose was 21 Gy(RBE) over 14 fractions as per the International Society of Paediatric Oncology European (SIOPEN) High-Risk-1 protocol for high-risk neuroblastoma [5]. In the case of 3D-CRT, ten patients exhibited a compromise on target volume coverage and/or a reduction in the total dose due to constraints on dose to nearby organs-at-risk (OAR), normally the kidneys. All IMAT and PBS-PT plans achieved full prescription delivery. Briefly, 3D-CRT plans were historically generated on Oncentra Masterplan® version 3.2 (Elekta) and clinically delivered. For one patient, the historical clinical doses could not be recovered so a reoptimized plan was used [11]. Most patients were treated with AP/PA fields; one was treated with a three-field technique. IMAT and PBS-PT were planned on Eclipse™ Version 13.7 (Varian Medical Systems). All IMAT plans consisted of dual arcs [11], while the PBS-PT plans consisted of two to four radiation fields, preferably posterior or posterior/oblique beams [15]. Plans were optimised and assessed according to the normal tissue constraints of the kidneys, liver, and vertebrae. Representative dose distribution maps and dose-volume histograms are shown in Fig. 1. Further information on key dose metrics (mean dose, V_2.5Gy_ and V_10Gy_) for SMN-relevant organs can be found in the Supplementary Material (Fig. S1).

Three-dimensional radiotherapy doses exported from the treatment planning systems (TPSs) were analysed. For PBS-PT, an analytical estimation of homogeneous whole-body neutron dose was added to the dose distributions for SMN risk estimation [17] based on published measurement data [31].

### 2.4 Estimation of second malignant neoplasm risk

The SMN risk was quantified through the concepts of Organ Equivalent Dose (OED) and Lifetime Attributable Risk (LAR).

The risk ratio (RR) between two modalities was estimated as the ratio of OEDs (Eq. (1)), a concept introduced by Schneider et al [27]. For example, the RR between PBS-PT and IMAT was defined as:

(1) $$RR_{PBS-PT/IMAT}=\frac{{OED}_{PBS-PT}}{{OED}_{IMAT}}$$

Schneider et al. proposed OED as a dose quantity proportional to the probability of carcinogenesis even for non-linear dose–response relationships and highly inhomogeneous dose distributions [27]. It was therefore quantified as the local dose weighted with a dose–response relationship function, averaged across the whole organ volume. We derived the OED from the total organ volume ($V_{T}$), the 3D dose map ($V\left(D_{i}\right)$), and the chosen dose–response relationship (RED) for SMN induction (Eq. (2)):

(2) $$OED=\frac{1}{V_{T}}\sum_{i}V\left(D_{i}\right)\times RED\left(D_{i}\right)$$

**Fig. 2:**
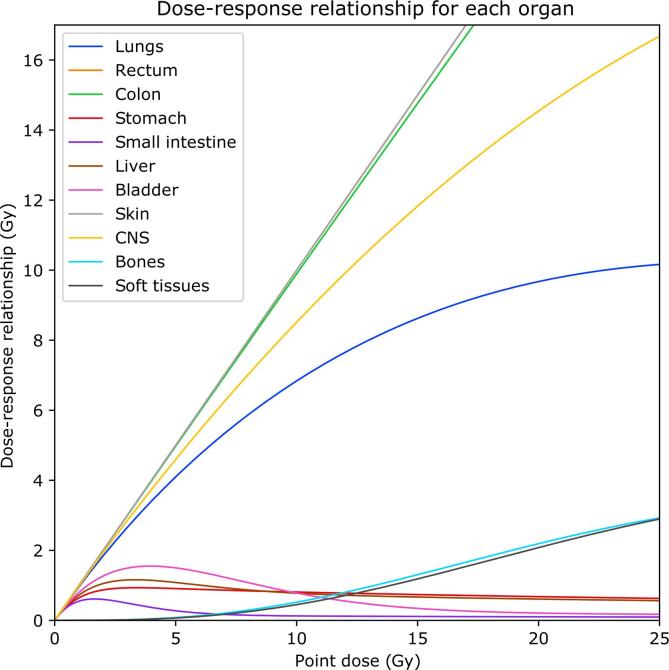
Dose-response relationship for the eleven organs considered. Note that the rectum and skin curves overlap since both used a linear dose–response relationship. Colour version online.

Several dose–response relationships were investigated by Schneider et al. in an effort to fit the historical data of the Atomic Bomb survivors [32] to the cancer risk data of Hodgkin’s disease patients [33]: the linear, bell-shaped, plateau, and full mechanistic models. The linear relationship assumes a linear response across the whole dose range and is defined solely by the total dose. The non-linear dose–response relationships account for cell death and repopulation between fractions and exhibit exponential changes in risk with increasing dose. The intermediate repopulation model was used for sarcoma induction in the bone and soft tissues. The full mechanistic model for carcinoma induction was used on all other organs, except for the rectum and skin where this model did not converge – a linear dose–response relationship was used instead. The ratio of mean doses between modalities was also calculated for all organs, to illustrate the impact of linear and non-linear dose-response relationships on SMN risk modelling. Fig. 2 shows all dose–response curves; complete details of equations and parameters are shown in the Supplementary Material (Table S1).

The Lifetime Attributable Risk (LAR) was defined as an individual’s cumulative probability of cancer incidence in excess of the baseline risk attributable to radiation exposure. It was calculated as the integral of the Excess Absolute Risk (EAR) over the expected lifespan (Eq. (3)) [16], [25], [26], [34]:

(3) $$\mathrm{LAR}\left(D,e,a,s\right)=\int_{a=e+L}^{\max(a)}EAR\left(D,e,a,s\right)\times \frac{S\left(a\right)}{S\left(e\right)}da$$

with the EAR adjusted to population statistics through the survival probability ratio (S(a)/S(e)), the conditional probability of an individual being alive at age a after radiation exposure at age e, according to UK population lifetables [35]. The maximum age attained (max(a)) and the latency period for solid cancer induction (L) were set as 75 and 5 years, respectively [25], [26]. The EAR itself was derived from the OED, the population dependent modifying function (μ(e,a)), the initial gradient of the dose–response curve ($\beta^{*}$), and the gender-specific factor (s, 0.17 for females and −0.17 for males) (Eq. (4)) [16], [25], [26], [27], [34]:

(4) $$EAR\left(D,e,a,s\right)=OED\times \mu \left(e,a\right)\times \beta^{*}\times \left(1+s\right)$$

with $\mu \left(e,a\right)=\exp\left(\gamma_{e}\left(e-30\right)+\gamma_{a}\ln\left(a/70\right)\right)$ defined from the age at exposure (e), attained age (a), and two organ-specific age-modifying parameters ($\gamma_{e}$ and $\gamma_{a}$) [32].

The cumulative LAR was calculated as the sum of the LARs of different organs, assuming that the LARs for different organs were independent of each other [26].

### 2.5 Data analysis

The OED, EAR and LAR for each organ and radiotherapy modality were calculated and analysed for all patients using MATLAB 2019a (MathWorks Inc). Statistical analysis was performed using the Wilcoxon signed-rank test at 5% significance level.

## 3 Results

**Fig. 3:**
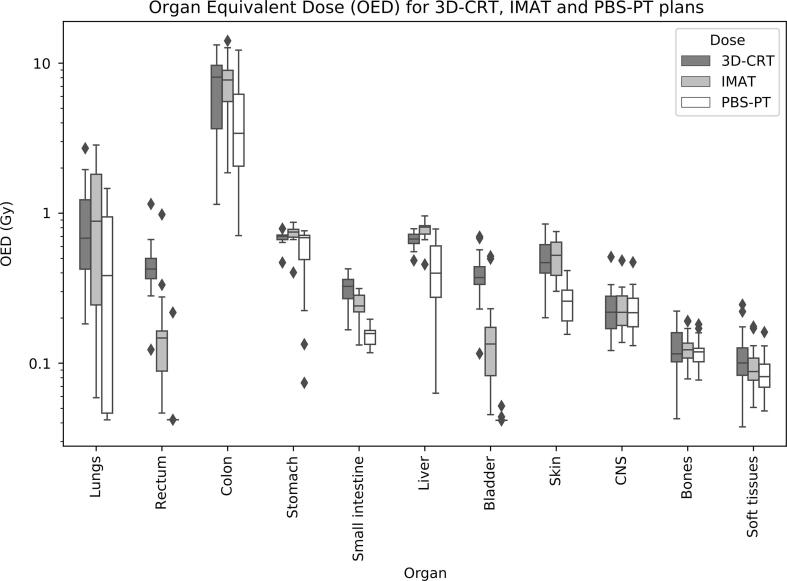
Boxplots of organ equivalent doses (OEDs) for 3D-CRT, IMAT and PBS-PT plans. Outliers (diamonds) fall outside the ± 2.7std range.

The OEDs calculated were overall the smallest for PBS-PT plans. For 3D-CRT the OEDs were generally comparable to IMAT plans, even lower for a few organs (such as liver and stomach). Complete data on the distribution of the OEDs per modality for the eleven organs studied can be seen in Fig. 3.

**Fig. 4:**
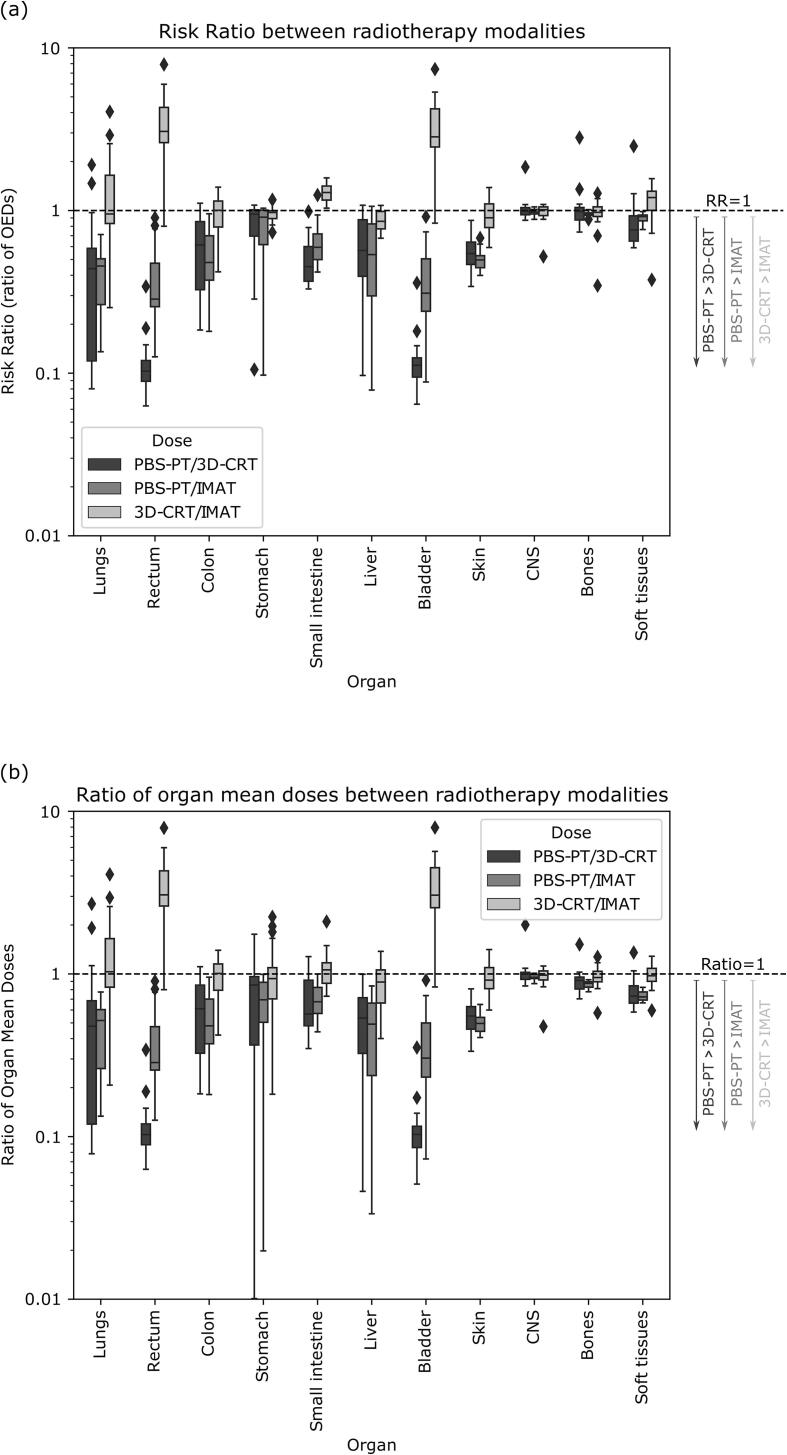
Boxplot of (a) the risk ratios (RRs) and (b) ratio of mean organ doses between modalities for all subjects included. Outliers (diamonds) fall outside the ± 2.7std range. Note that the data is similar in both plots for rectum and skin, as their organ equivalent doses (OED) were also calculated using a linear dose–response relationship.

Overall, the estimated risk of SMN was reduced in PBS-PT vs 3D-CRT and IMAT, such that $RR_{PBS-PT/IMAT}$ and $RR_{PBS-PT/3D-CRT}$ were < 1 for most organs and patients. Per organ, the $RR_{PBS-PT/IMAT}$ ranged from 0.38 ± 0.22 (bladder) to 0.98 ± 0.04 (CNS), while $RR_{PBS-PT/3D-CRT}$ ranged from 0.12 ± 0.06 (rectum and bladder) to 1.06 ± 0.43 (bone). Considering IMAT and 3D-CRT, $RR_{3D-CRT/IMAT}$ was on average closer to one, without a clear trend across all organs and patients on which modality was superior. The distributions of the ratios of OEDs and mean doses for all organs and modalities are shown in Fig. 4. In general, similar trends were found for the ratio of OEDs and mean organ doses, with the benefits of PBS-PT being clear with both linear and non-linear dose–response relationships. When considering linear dose–response relationship to compare PBS-PT with 3D-CRT and IMAT, stronger benefits of PBS-PT were estimated for the stomach, liver, bone, and soft tissues, while the lungs and small intestine showed less benefits. With linear dose-responses 3D-CRT and IMAT risks become more comparable overall. Full data shown in Supplementary Material (Table S2).

No strong statistically significant differences were found in organ-specific RRs between PBS-PT and photon-modalities when the patient group was split into two according to: tumour position (midline vs unilateral, midline and bilateral vs other [15]), number of PBS-PT beams (2 vs > 2 beams), age (below vs greater than median age), and PTV size (below vs greater than median size). This indicated a similar degree of benefit in PBS-PT across the patient group.

**Fig. 5:**
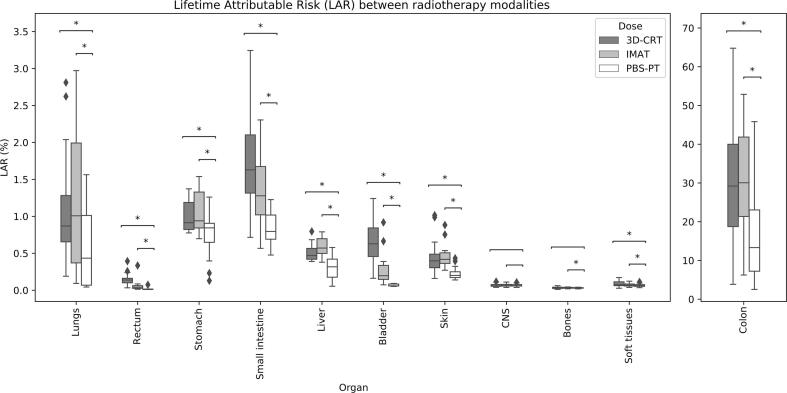
Boxplot of the Lifetime Attributable Risk (LAR) for 3D-CRT, IMAT and PBS-PT plans. Asterisks indicate statistically significant results (p < 0.05, Wilcoxon signed-rank test). Outliers (diamonds) fall outside the ± 2.7std range.

The order of magnitude of the absolute risks (assessed using the LAR concept) for most organs ranged 0.01–1% and was similar between modalities (Fig. 5). PBS-PT was associated with statistically significant lower LAR values (p < 0.05, Wilcoxon signed-rank test) for all organs except the CNS (versus 3D-CRT/IMAT) and bone (versus 3D-CRT). The cumulative LARs were 21 ± 13% for PBS-PT, 35 ± 16% for 3D-CRT and 35 ± 14% for IMAT (3.0 ± 0.7%, 5.8 ± 1.4% and 5.2 ± 1.2% respectively, if excluding the colon). The difference in cumulative LAR between modalities was 14 ± 13% (PBS-PT vs 3D-CRT), 15 ± 8% (PBS-PT vs IMAT) and 0 ± 7% (3D-CRT vs IMAT). Full data shown in Supplementary Material (Table S3).

SMN risks and their implications varied between the different organs considered in analysis. The dosimetric benefits of PBS-PT (in terms of mean dose, V_2.5Gy_ and V_10Gy_) did not always translate into a favourable RR in the liver (for n={3,2} for 3D-CRT and IMAT, with mean liver doses of 4.4 ± 3.6 Gy(RBE), 8.4 ± 4.1 Gy and 7.6 ± 5.1 Gy for PBS-PT, IMAT and 3D-CRT, respectively), a consequence of the non-linear dose–response model for carcinogenesis used. In the skin, while the RR was always favourable to PBS-PT, the V_10Gy_ was greater in PBS-PT than IMAT, both lower than 3D-CRT (2.6 ± 1.0% vs 3.6 ± 1.5% vs 7.1 ± 3.7%). For the CNS, bone, and soft tissues the benefits of PBS-PT were insignificant or modest, with all modalities presenting similar OEDs. In fact, key dose metrics (mean dose, V_2.5Gy_ and V_10Gy_) of the CNS were also similar for 3D-CRT, IMAT and PBS-PT; V_10Gy_ was similar in the bone and soft tissues across all modalities. Despite lower average doses in comparison with other organs, the absolute risks estimated were high for the lungs and colon, a result of the combination of a greater increase in EAR both with increasing dose (Fig. 2) and attained age ($\gamma_{a}$ in Table S1) The rectum and bladder were peripheral to the radiation fields in most cases, and hence received generally low levels of radiation (V_2.5Gy_ < 1%).

## 4 Discussion

This study demonstrated the relative benefits of PBS-PT versus photon-based modalities (IMAT and 3D-CRT) in the treatment of paediatric abdominal neuroblastoma, using risk models of radiation-induced SMNs. Ours was the first study focused on abdominal neuroblastomas and state-of-the-art photon and proton modalities. It included a relatively large number of patients and detailed anatomical sub-volumes, where both relative and absolute risks were estimated using a method developed for SMN risk after therapeutic radiation exposures. The RRs and LARs reported were consistently favourable to PBS-PT plans, indicating that in terms of SMN risk there was a general benefit for abdominal neuroblastoma patients (not just for selected cases). IMAT was associated with a similar SMN risk to 3D-CRT. This was likely a consequence of the low dose radiation bath characteristic of IMAT. It is important to note that while 3D-CRT might appear superior to PBS-PT and IMAT in some cases, target coverage was limited in half the patient group due to other OAR constraints.

Others have also studied SMN risk in neuroblastoma treatment. However, to the best of our knowledge, studies as comprehensive as ours with state-of-the-art modalities (IMAT and PBS-PT) were not previously conducted. Fuji et al. investigated the SMN risk of 3D-CRT, Intensity Modulated Radiation Therapy (IMRT) and Passive Scattering Proton Therapy (PS-PT) in five paediatric neuroblastoma patients and observed the lowest LARs with PS-PT [16]. The estimated risk of SMN from PS-PT ranged 24–80% of that in 3D-CRT in the liver, stomach, bone, colon, and small intestine; IMRT was associated with similar risks to 3D-CRT. While not directly comparable, as different techniques were investigated, our estimated average risk of SMN from PBS-PT ranged 50–106% of 3D-CRT, with an extended patient selection. Hillbrand et al. evaluated the SMN risk in five neuroblastoma patients after 3D-CRT, IMRT, PS-PT, and PBS-PT, and reported a lifetime risk of SMN with PBS-PT of 0.6 ± 1.5% (considering risks in the liver, kidneys and grouping the remaining tissues) [17]. There were fundamental differences in their risk estimation methodology compared to our study, such as less detailed anatomical sub-volumes. Furthermore, dose–response relationships for primary radiation were based on the linear-quadratic model and linear risk coefficients listed by the ICRP 60 report [28]. Finally, Tamura et al. calculated the SMN LAR of a cohort of eight paediatric abdominal cancer patients, of which four were neuroblastomas [26]. They also employed Schneider’s mechanistic model and described lower LARs of SMN for PBS-PT compared to IMRT in the breast, lungs, colon, stomach, small intestine, liver, bladder, bone, and soft tissues. Although absolute LARs were not provided, Tamura et al. reported a cumulative LAR difference of 16.6 ± 19.9% between IMRT and PBS-PT in abdominal cases, led by differences in the colon, as found in this study (12.4 ± 7.7% between IMAT and PBS-PT reported here).

Our study highlighted the complementary value of SMN risk metrics when comparing rival treatment modalities, as well as the importance of analysing both absolute and relative risks for detailed anatomical sub-volumes. The benefits measured using traditional dosimetric quantities, such as mean dose, did not necessarily translate into favourable SMN risks for non-linear dose responses for individual patients and organs. The dose–response relationships and age dependencies varied across organs, which were planned to receive doses ranging from background to therapeutic doses – all these aspects must be carefully considered and balanced. Although, the gastrointestinal tract is often merged for planning optimisation purposes, the RRs and LARs reported differed substantially for its sub-volumes and so merited independent assessment. The skin doses achieved in PBS-PT were beneficial in terms of SMN risk, but at the cost of larger volumes receiving 10 Gy or more, which may become important when considering other acute and late effects in the skin. The vertebrae adjacent to the target volume are homogeneously irradiated in paediatric patients to reduce growth asymmetry. Thus, the CNS was partially inside the target volume which resulted in insignificant differences in SMN risk between modalities for this organ. For bone and soft tissues, the modest benefits of PBS-PT reported might appear counter-intuitive considering the theoretical tissue-sparing capabilities recognised in PBS-PT. However, one must consider that the dosimetric differences between modalities were more pronounced at lower doses levels where the dose-response used for sarcoma induction was weak. The lungs typically received low mean doses compared to other organs. However, as they are particularly radiosensitive, they were linked to absolute risks of similar magnitude. The bladder and rectum were distant to the target; as such, the RR values reported were thus associated with larger uncertainties in out-of-field dose calculations between modalities.

In general, PBS-PT appeared beneficial from a SMN risk point-of-view. However, this is only one of the aspects to consider during childhood cancer management. The best plan dosimetrically should always be chosen considering coverage and robustness, as well as other acute and late effects. IMAT was previously found to be preferable to PBS-PT on 15% of the cases, as gas in the gastrointestinal tract degraded the quality of PBS-PT plans, particularly in midline tumours [9].

We reported LARs with orders of magnitude in the range of 0.01–1% for most organs and tissues (except the colon). Cumulative cancer incidence in neuroblastoma survivors after 20 years was quoted as 1.9% in the Childhood Cancer Survivor Study (CCSS) [23], and 2.2% from French and British institutions using historical radiotherapy techniques [22]. However, a direct comparison of our findings to historical follow-up data was very challenging; differences in radiation delivery and other factors (such as genetic susceptibility and combination with other therapeutic interventions) confound the inherent risks.

This study had certain limitations. Firstly, the dose maps used were exported from the TPS, and as such, larger uncertainties were associated with doses to organs more distant from the target (such as bladder and rectum) due to secondary radiation (produced before and after entering the patient). Accurately measuring peripheral dose is a challenging problem, and TPSs are not usually commissioned to accurately model these dose levels [36]. Furthermore, different TPSs were used for 3D-CRT and IMAT plans, which also impacted peripheral dose calculations. In the case of PBS-PT, the TPS used assigns zero dose a few centimetres outside the field margins. Thus, to avoid substantially overestimating PBS-PT benefits for out-of-field organs, a background neutron dose was added in the analyses [31]. Nevertheless, Monte Carlo simulations would be required to better model the risks in these regions [37], [38].

Incomplete volumes for the CNS, skin, bone and soft tissues were also a limitation. For example, if naively calculated using CT information only, the LAR in the CNS for PBS-PT would have been calculated as 2.0 ± 0.5% versus 0.07 ± 0.02% with XCAT information, as the irradiated spine only comprised a fraction of the whole CNS. Our methodology accounted for such gross errors, but uncertainties were still associated with the quality of the matching between each subject and their template anatomy. Furthermore, the quality of the alignment varied with similarity of positioning and body mass index of each patient with the default XCAT phantoms.

Finally, there were also uncertainties associated with the methodology used to convert radiation exposure into relative and absolute risk of radiation-induced SMNs. Our study used the methodology developed by Schneider et al. that was established for therapeutic exposures and accounts for the effects of cell killing and treatment fractionation. However, it was optimised using data from atomic bomb survivors and Hodgkin’s disease patients [32], [33], populations which are inherently different from paediatric neuroblastoma patients in terms of radiation exposure, genetic susceptibility, and age at exposure. Alternative risk models were developed in the context of therapeutic exposures to radiation [28], [39], as well as for low dose occupational exposures [40], [41]. However, these are also associated with limitations and uncertainties. For example, SMN excess relative risks (per Gy) after radiotherapy are smaller than for low-dose exposures [42], therefore, applying linear risk models developed for acute low-dose exposures to therapeutic settings tends to inflate risk predictions [43].

The true shape of the dose–response relationship model at higher doses is still a source of debate. Larger studies have not found clear evidence of non-linear dose response relationships in the direction of a downturn in risk, with the exception of thyroid cancer [42]. However, the statistical power of the analysis was likely limited for detecting non-linearity. In our study we found similar trends in relative risk using linear and non-linear dose–response relationships, with the latter being more conservative on estimating the benefits of PBS-PT. This is in agreement with the general consensus that uncertainties are less pronounced when reporting relative risks [27], and that absolute risks should be interpreted with caution.

To conclude, PBS-PT was associated with the lowest risk of radiation-induced SMN compared to IMAT and 3D-CRT in abdominal neuroblastoma treatment. However, the best plan should also consider other clinical endpoints and plan robustness. Further studies must be conducted to verify the overall dosimetric advantages of PBS-PT clinically and inform optimal patient selection.

## Declaration of Competing Interest

The authors declare that they have no known competing financial interests or personal relationships that could have appeared to influence the work reported in this paper.

## References

[1] National Cancer Registration and Analysis Service. Incidence of Cancer in Teenagers and Young Adults Report 2021. http://www.ncin.org.uk/cancer_type_and_topic_specific_work/cancer_type_specific_work/cancer_in_children_teenagers_and_young_adults/ (accessed May 20, 2021).

[2] Matthay K.K., Maris J.M., Schleiermacher G., Nakagawara A., Mackall C.L., Diller L. (2016). Neuroblastoma Nat Rev Dis Prim.

[3] Sung K.W., Yoo K.H., Koo H.H., Kim J.Y., Cho E.J., Seo Y.L. (2009). Neuroblastoma originating from extra-abdominal sites: association with favorable clinical and biological features. J Korean Med Sci.

[4] Arumugam S., Manning-Cork N.J., Gains J.E., Boterberg T., Gaze M.N. (2019). The evidence for external beam radiotherapy in high-risk neuroblastoma of childhood: a systematic review. Clin Oncol.

[5] Pinto N.R., Applebaum M.A., Volchenboum S.L., Matthay K.K., London W.B., Ambros P.F. (2015). Advances in risk classification and treatment strategies for neuroblastoma. J Clin Oncol.

[6] Tas M.L., Reedijk A.M.J., Karim-Kos H.E., Kremer L.C.M., van de Ven C.P., Dierselhuis M.P. (2020). Neuroblastoma between 1990 and 2014 in the Netherlands: Increased incidence and improved survival of high-risk neuroblastoma. Eur J Cancer.

[7] Ladenstein R., Pötschger U., Pearson A.D.J., Brock P., Luksch R., Castel V. (2017). Busulfan and melphalan versus carboplatin, etoposide, and melphalan as high-dose chemotherapy for high-risk neuroblastoma (HR-NBL1/SIOPEN): an international, randomised, multi-arm, open-label, phase 3 trial. Lancet Oncol.

[8] Holmes K., Pötschger U., Pearson A.D.J., Sarnacki S., Cecchetto G., Gomez-Chacon J. (2020). Influence of surgical excision on the survival of patients with stage 4 high-risk neuroblastoma: A report from the HR-NBL1/SIOPEN study. J Clin Oncol.

[9] Ladenstein R., Pötschgerulrike U., Valteau-Couanet D., Luksch R., Castel V., Ash S. (2020). Investigation of the role of dinutuximab beta-based immunotherapy in the siopen high-risk neuroblastoma 1 trial (HR-NBL1). Cancers (Basel).

[10] Gaze M.N., Boterberg T., Dieckmann K., Hörmann M., Gains J.E., Sullivan K.P. (2013). Results of a quality assurance review of external beam radiation therapy in the international society of paediatric oncology (Europe) neuroblastoma group’s high-risk neuroblastoma trial: a SIOPEN study. Int J Radiat Oncol Biol Phys.

[11] Gains J.E., Stacey C., Rosenberg I., Mandeville H.C., Chang Y.-C., D’Souza D. (2013). Intensity-modulated arc therapy to improve radiation dose delivery in the treatment of abdominal neuroblastoma. Future Oncol.

[12] Gains J., Mandeville H., Cork N., Brock P., Gaze M. (2012). Ten challenges in the management of neuroblastoma. Future Oncol.

[13] Laverdière C., Cheung N.-K., Kushner B.H., Kramer K., Modak S., LaQuaglia M.P. (2005). Long-term complications in survivors of advanced stage neuroblastoma. Pediatr Blood Cancer.

[14] Lim P.S., Pica A., Hrbacek J., Bachtiary B., Walser M., Lomax A.J. (2020). Pencil beam scanning proton therapy for paediatric neuroblastoma with motion mitigation strategy for moving target volumes. Clin Oncol.

[15] Lim P.S., Rompokos V., Bizzocchi N., Gillies C., Gosling A., Royle G. (2021). Pencil beam scanning proton therapy case selection for paediatric abdominal neuroblastoma: effects of tumour location and bowel gas. Clin Oncol.

[16] Fuji H., Schneider U., Ishida Y., Konno M., Yamashita H., Kase Y. (2013). Assessment of organ dose reduction and secondary cancer risk associated with the use of proton beam therapy and intensity modulated radiation therapy in treatment of neuroblastomas. Radiat Oncol.

[17] Hillbrand M., Georg D., Gadner H., Pötter R., Dieckmann K. (2008). Abdominal cancer during early childhood: a dosimetric comparison of proton beams to standard and advanced photon radiotherapy. Radiother Oncol.

[18] Hill-Kayser C., Tochner Z., Both S., Lustig R., Reilly A., Balamuth N. (2013). Proton versus photon radiation therapy for patients with high-risk neuroblastoma: the need for a customized approach. Pediatr Blood Cancer.

[19] Hall E.J. (2006). Intensity-modulated radiation therapy, protons, and the risk of second cancers. Int J Radiat Oncol Biol Phys.

[20] Armstrong G.T., Liu Q.i., Yasui Y., Neglia J.P., Leisenring W., Robison L.L. (2009). Late mortality among 5-year survivors of childhood cancer: a summary from the childhood cancer survivor study. J Clin Oncol.

[21] Bhatia S., Constine L.S. (2009). Late morbidity after successful treatment of children with cancer. Cancer J.

[22] Rubino C., Adjadj E., Guérin S., Guibout C., Shamsaldin A., Dondon M.-G. (2003). Long-term risk of second malignant neoplasms after neuroblastoma in childhood: role of treatment. Int J Cancer.

[23] Neglia J.P., Friedman D.L., Yasui Y., Mertens A.C., Hammond S., Stovall M. (2001). Second malignant neoplasms in five-year survivors of childhood cancer: childhood cancer survivor study. J Natl Cancer Inst.

[24] Ng J., Shuryak I. (2014). Minimizing second cancer risk following radiotherapy: current perspectives. Cancer Manage Res.

[25] Moteabbed M., Yock T.I., Paganetti H. (2014). The risk of radiation-induced second cancers in the high to medium dose region: a comparison between passive and scanned proton therapy, IMRT and VMAT for pediatric patients with brain tumors. Phys Med Biol.

[26] Tamura M., Sakurai H., Mizumoto M., Kamizawa S., Murayama S., Yamashita H. (2017). Lifetime attributable risk of radiation-induced secondary cancer from proton beam therapy compared with that of intensity-modulated X-ray therapy in randomly sampled pediatric cancer patients. J Radiat Res.

[27] Schneider U., Sumila M., Robotka J. (2011). Site-specific dose-response relationships for cancer induction from the combined Japanese A-bomb and Hodgkin cohorts for doses relevant to radiotherapy. Theor Biol Med Model.

[28] Daşu A., Toma-Daşu I., Olofsson J., Karlsson M. (2005). The use of risk estimation models for the induction of secondary cancers following radiotherapy. Acta Oncol.

[29] Segars W.P., Norris H., Sturgeon G.M., Zhang Y., Bond J., Minhas A. (2015). The development of a population of 4D pediatric XCAT phantoms for imaging research and optimization. Med Phys.

[30] Norris H., Zhang Y., Bond J., Sturgeon G.M., Minhas A., Tward D.J. (2014). A set of 4D pediatric XCAT reference phantoms for multimodality research. Med Phys.

[31] Schneider U., Agosteo S., Pedroni E., Besserer J. (2002). Secondary neutron dose during proton therapy using spot scanning. Int J Radiat Oncol Biol Phys.

[32] Preston D.L., Ron E., Tokuoka S., Funamoto S., Nishi N., Soda M. (2007). Solid cancer incidence in atomic bomb survivors: 1958–1998. Radiat Res.

[33] Dores G.M., Metayer C., Curtis R.E., Lynch C.F., Clarke E.A., Glimelius B. (2002). Second malignant neoplasms among long-term survivors of Hodgkin’s disease: a population-based evaluation over 25 years. J Clin Oncol.

[34] Sakthivel V., Ganesh K.M., McKenzie C., Boopathy R., Selvaraj J. (2019). Second malignant neoplasm risk after craniospinal irradiation in X-ray-based techniques compared to proton therapy. Australas Phys Eng Sci Med.

[35] Office for National Statistics. National life tables, UK: 2015 to 2017. Stat Bull 2018:1–11. https://www.ons.gov.uk/peoplepopulationandcommunity/birthsdeathsandmarriages/lifeexpectancies/bulletins/nationallifetablesunitedkingdom/2015to2017.

[36] Takam R., Bezak E., Marcu L.G., Yeoh E. (2011). Out-of-field neutron and leakage photon exposures and the associated risk of second cancers in high-energy photon radiotherapy: current status. Radiat Res.

[37] Yeom Y.S., Kuzmin G., Griffin K., Mille M., Polf J., Langner U. (2020). A monte carlo model for organ dose reconstruction of patients in pencil beam scanning (PBS) proton therapy for epidemiologic studies of late effects. J Radiol Prot.

[38] Huang J.Y., Followill D.S., Wang X.A., Kry S.F. (2013). Accuracy and sources of error of out-of field dose calculations by a commercial treatment planning system for inte sity-modulated radiation therapy treatments. J Appl Clin Med Phys.

[39] Shuryak I., Hahnfeldt P., Hlatky L., Sachs R.K., Brenner D.J. (2009). A new view of radiation-induced cancer: integrating short- and long-term processes. Part II: Second cancer risk estimation. Radiat Environ Biophys.

[40] National Research Council (2006). Health risks from exposure to low levels of ionizing radiation: BEIR VII Phase 2.

[41] International Commission on Radiological Protection (2007). The 2007 Recommendations of the International Commission on Radiological Protection. ICRP publication 103. Ann ICRP.

[42] Berrington de Gonzalez A., Gilbert E., Curtis R., Inskip P., Kleinerman R., Morton L. (2013). Second solid cancers after radiation therapy: a systematic review of the epidemiologic studies of the radiation dose-response relationship. Int J Radiat Oncol Biol Phys.

[43] Trott K.R. (2017). Special radiobiological features of second cancer risk after particle radiotherapy. Phys Medica.

