# Supplementary material for: Risk of radiation-induced second malignant neoplasms from photon and proton radiotherapy in paediatric abdominal neuroblastoma
Source: Phys Imaging Radiat Oncol. 2021 Jul 9;19:45–52. doi: 10.1016/j.phro.2021.06.003 (PMC8295851; doi:10.1016/j.phro.2021.06.003)
Supplement: Supplementary data 1 [file mmc1.docx]

**SUPPLEMENTARY MATERIAL**


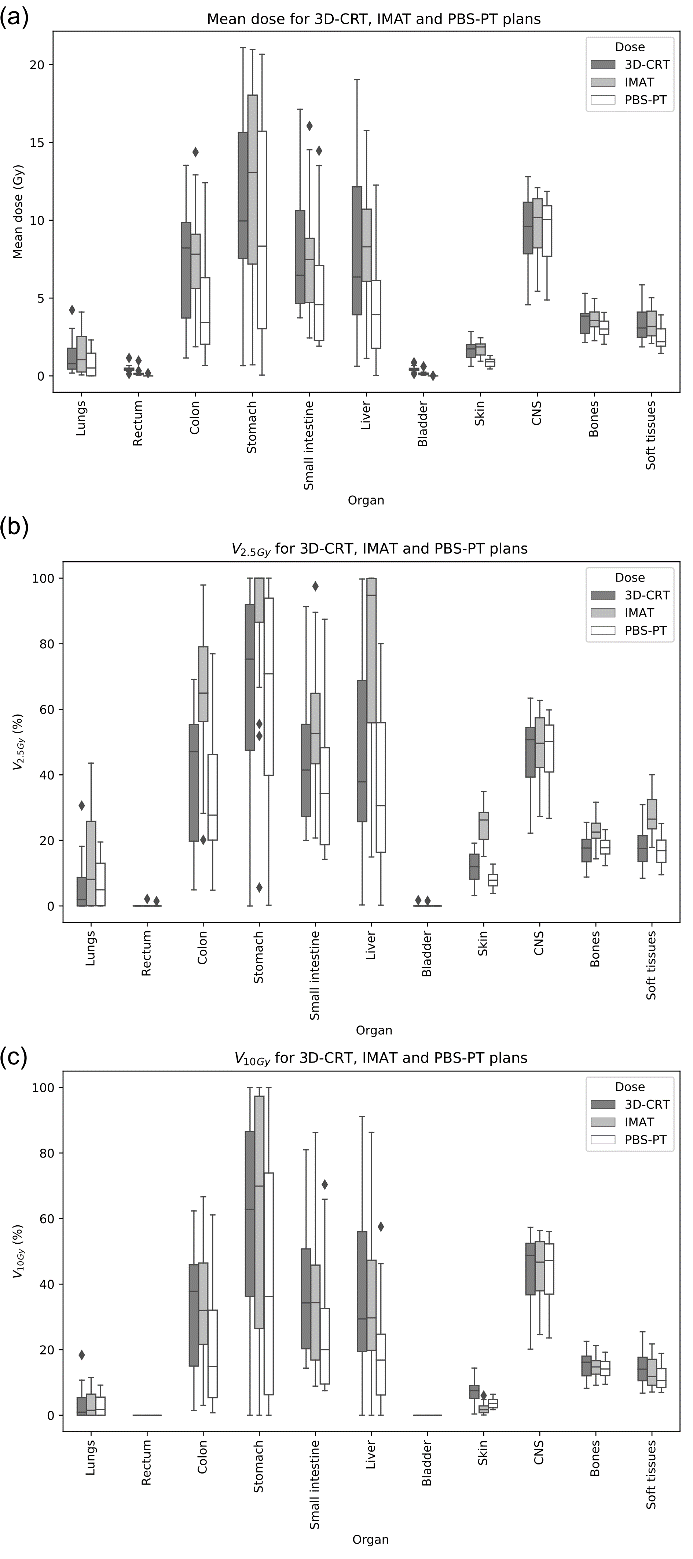


Figure S.1- Key dose metrics of 3D-CRT, IMAT, and PBS-PT plans for SMN-relevant organs: (a) mean doses, (b) relative volume receiving 2.5Gy, and (c) 10Gy or more. Measures provided without considering out-of-field anatomy with phantom or neutron dose contributions to PBS-PT plans. Notice how V_2.5Gy_=100% for n={11,4} subjects in the stomach and liver with IMAT. Outliers (diamonds) fall outside the ±2.7std range.

Table S1- Organ-specific dose-response relationships and parameters.

| Organ | Dose-response relationship (RED) model+ | β*  (104 PY Gy-1) | $\alpha$  (Gy-1) | R | $\gamma_{e}$ | $\gamma_{a}$ |
| --- | --- | --- | --- | --- | --- | --- |
| Lungs | Carcinoma Mechanistic | 8.0$ | 0.042 | 0.83 | 0.002 | 4.23 |
| Rectum | Carcinoma Linear | 0.73$^#^ | NA | NA | -0.024 | 2.38 |
| Colon | Carcinoma Mechanistic | 7.4$ | 0.001 | 0.99 | -0.056 | 6.9 |
| Stomach | Carcinoma Mechanistic | 5.2$ | 0.460 | 0.46 | -0.002 | 1.9 |
| Small intestine | Carcinoma Mechanistic | 10$ | 0.591 | 0.09 | -0.056 | 6.9 |
| Liver | Carcinoma Mechanistic | 2.4$ | 0.323 | 0.29 | -0.021 | 3.6 |
| Bladder | Carcinoma Mechanistic | 3.8$ | 0.219 | 0.06 | -0.024 | 2.38 |
| Skin | Carcinoma Linear | 1.1 | NA | NA | -0.061 | 4.36 |
| CNS | Carcinoma Mechanistic | 0.70$ | 0.018 | 0.93 | -0.024 | 2.38 |
| Bone | Sarcoma Mechanistic | 0.20 | 0.067 | 0.5 | -0.013 | -0.56 |
| Soft tissue | Sarcoma Mechanistic | 0.60 | 0.060 | 0.5 | -0.013 | -0.56 |
| +Dose-response relationship (RED) models from Schneider et al^[[1]](#footnote-1)^:   - Carcinoma linear model: $\mathrm{RED}\left( D \right)=D$ - Carcinoma full mechanistic model: $\mathrm{RE}D_{\mathrm{carcinoma}}\left( D \right)=\frac{e^{-\alpha^{'}D}}{\alpha^{'}R}\left( 1-2R+R^{2}e^{\alpha^{'}D}-\left( 1-R \right)^{2}e^{-\frac{\alpha^{'}R}{1-R}D} \right)$ - Sarcoma full mechanistic model: $\mathrm{RE}D_{\mathrm{sarcoma}}\left( D \right)=\frac{e^{-\alpha^{'}D}}{\alpha^{'}R}\left( 1-2R+R^{2}e^{\alpha^{'}D}-\left( 1-R \right)^{2}e^{-\frac{\alpha^{'}R}{1-R}D}-\alpha'RD \right)$   where:   - $D$ is the total dose - R is the repair/repopulation parameter - $\alpha^{'}$ is the cell killing factor, defined by the linear quadratic model as $\alpha^{'}=\alpha+\beta\frac{D}{D_{T}}d_{T}$ with $\alpha/\beta=3$ - $D_{T}$ is the prescribed dose to the target volume - $d_{T}$ is the prescribed dose per fraction   $β* equals $\beta_{\mathrm{EAR}}$ transferred to the UK population from Preston et al^[[2]](#footnote-2)^ for mechanistic dose-response relationships.  ^#^ the linear fit for the rectum did not converge in Schneider et al therefore the linear parameters used were from Preston et al. | | | | | | |

Table S2- Risk ratio (RR) between radiotherapy modalities for the eleven organs considered in this study. The ratios were calculated using both the concept of Organ Equivalent Dose and Mean Organ Dose, corresponding to non-linear and linear dose-response curves for second malignant neoplasm induction, respectively.

|  | Ratios between radiotherapy modalities | | | | | |
| --- | --- | --- | --- | --- | --- | --- |
|  | Risk Ratio | | | Ratio of Mean Organ Doses | | |
| Organ | PBS-PT/  IMAT | PBS-PT/  3D-CRT | 3D-CRT/  IMAT | PBS-PT/  IMAT | PBS-PT/  3D-CRT | 3D-CRT/  IMAT |
| Lungs | 0.41±0.16 | 0.51±0.48 | 1.36±0.96 | 0.45±0.20 | 0.61±0.67 | 1.39±0.97 |
| Rectum^+^ | 0.39±0.22 | 0.12±0.06 | 3.50±1.75 | 0.39±0.22 | 0.12±0.06 | 3.50±1.75 |
| Colon | 0.53±0.22 | 0.60±0.31 | 0.97±0.27 | 0.53±0.22 | 0.60±0.31 | 0.97±0.27 |
| Stomach | 0.78±0.30 | 0.81±0.29 | 0.95±0.11 | 0.65±0.30 | 0.77±0.44 | 1.02±0.52 |
| Small intestine | 0.65±0.21 | 0.50±0.17 | 1.30±0.16 | 0.69±0.17 | 0.69±0.27 | 1.09±0.32 |
| Liver | 0.55±0.31 | 0.61±0.31 | 0.87±0.13 | 0.46±0.25 | 0.53±0.26 | 0.86±0.29 |
| Bladder | 0.38±0.22 | 0.12±0.06 | 3.23±1.55 | 0.38±0.22 | 0.11±0.06 | 3.44±1.66 |
| Skin^+^ | 0.50±0.07 | 0.56±0.15 | 0.94±0.22 | 0.50±0.07 | 0.55±0.14 | 0.98±0.21 |
| CNS | 0.98±0.04 | 1.03±0.20 | 0.98±0.12 | 0.96±0.04 | 1.03±0.24 | 0.96±0.14 |
| Bone | 0.95±0.03 | 1.06±0.43 | 0.96±0.19 | 0.86±0.04 | 0.92±0.17 | 0.97±0.14 |
| Soft tissues | 0.90±0.05 | 0.88±0.42 | 1.14±0.29 | 0.74±0.05 | 0.78±0.18 | 0.98±0.16 |
| ^+^the linear dose-response relationship was used for the OED calculation in the rectum and skin; therefore, the two ratios are equal. | | | | | | |

Table S3- Lifetime Attributable Risk (LAR) between radiotherapy modalities for the eleven organs considered in this study.

|  | Lifetime Attributable Risk (LAR) | | |
| --- | --- | --- | --- |
| Organ | 3D-CRT | IMAT | PBS-PT |
| Lungs | 1.1±0.7 | 1.2±1.0 | 0.6±0.5 |
| Rectum | 0.15±0.08 | 0.06±0.07 | 0.02±0.01 |
| Colon | 29±15 | 30±14 | 18±13 |
| Stomach | 1.0±0.2 | 1.1±1.1 | 0.8±0.3 |
| Small intestine | 1.8±0.6 | 1.4±0.5 | 0.8±0.2 |
| Liver | 0.5±0.1 | 0.6±0.1 | 0.3±0.2 |
| Bladder | 0.66±0.27 | 0.26±0.21 | 0.07±0.01 |
| Skin | 0.4±0.2 | 0.5±0.1 | 0.2 ±0.1 |
| CNS | 0.07±0.02 | 0.07±0.02 | 0.07±0.02 |
| Bone | 0.03±0.01 | 0.03±0.01 | 0.03±0.01 |
| Soft tissues | 0.90±0.04 | 0.07±0.02 | 0.07±0.02 |
| Cumulative | 35±16 | 35±14 | 21±13 |

1. <https://doi.org/10.1186/1742-4682-8-27> [↑](#footnote-ref-1)
2. <https://doi.org/10.1667/RR0763.1> [↑](#footnote-ref-2)
